# Supplementary material for: Genome-Wide Identification and Analysis of the WRKY Gene Family and Cold Stress Response in Acer truncatum
Source: Genes (Basel). 2021 Nov 24;12(12):1867. doi: 10.3390/genes12121867 (PMC8701280; doi:10.3390/genes12121867)
Supplement: Supplementary file 1 [file genes-12-01867-s001.zip › Table S2.pdf]

genes

Table S2. *AtruWRKY* gene-specific primers.

ID	*AtruWRKY* gene name	Primer sequences (5' to 3')	
		F	R
1	*AtruWRKY11*	ATCAACAGACCCTAAAGCAGTC	TGTTCCTGAAATCTGTCCTGG
2	*AtruWRKY12*	GTGGAGTCCTTAATCACGAGG	TCTTGAGTGACATGGTGCG
3	*AtruWRKY13*	GGTTGCACATTTTCCCTTGG	CCTATCCCCATCCCCATTTTAA
4	*AtruWRKY15*	GGTTTGTGGGTGAGTAGGTG	ACTTTCTTGCCGTACTTCCTC
5	*AtruWRKY17*	TCAGGTTCAAAGGTGGATGG	GGGAAAACCAGCCATTGATG
6	*AtruWRKY20*	TCTCGGCAATGTTAGGTGATG	ACCCGTTTCCTTTCTGTCAG
7	*AtruWRKY25*	TCAAGAATTCAGATCTCGCCG	GAAGTAGAAGCAGAAGGAGACG
8	*AtruWRKY28*	CTCCCAAGTCTTTCTCTTCCAG	AGCCTGGAACATCCTGAAAG
9	*AtruWRKY29*	AGAAGTGTCGAAGACCAATCG	TTTGAGAAGGGTGTGGATGG
10	*AtruWRKY31*	AGCACGTAGAAAGAGCATCAG	CTTTACTAGCAGGGACCGATG
11	*AtruWRKY33*	TCTTGGCCGGAAAATATCTCC	GAGTTGGAGAGCGAAGTCAC
12	*AtruWRKY39*	GGTGGACAGATAAAGTGAGAGTC	CTCTACCTGGGATATTTGGCTG
13	*AtruWRKY44*	CAACGTGAAATCGAAGCACC	ACAAGACAAAGACAAGCCTCC
14	*AtruWRKY47*	AGTTAAGAACAACAAATTTCCCAGG	GTTGGCCCTTGTTTCTTTGG
15	*AtruWRKY51*	CAGGAGCTATTTCAAGTGTCAAAG	CCTAATCTCACCCAACTCACC
16	18S	GAGGTAGCTTCGGGCGCAACT	GCAGGTTAGCGAAATGCGATAC
